# Phospholipid Complex Technique for Superior Bioavailability of Phytoconstituents

**DOI:** 10.15171/apb.2017.005

**Published:** 2017-04-13

**Authors:** Kattamanchi Gnananath, Kalakonda Sri Nataraj, Battu Ganga Rao

**Affiliations:** ^1^Department of Pharmaceutical Analysis, Shri Vishnu College of Pharmacy, Vishnupur, Bhimavaram-534202, Andhra Pradesh, India.; ^2^Department of Pharmacognosy, University College of Pharmaceutical Sciences, Vishakhapatnam-530003, Andhra Pradesh, India.

**Keywords:** Phytoconstituents, NDDS, Phospholipids, Phytosome, Bioavailability

## Abstract

Phytoconstituents have been utilized as medicines for thousands of years, yet their application is limited owing to major hurdles like deficit lipid solubility, large molecular size and degradation in the gastric environment of gut. Recently, phospholipid-complex technique has unveiled in addressing these stumbling blocks either by enhancing the solubilizing capacity or its potentiating ability to pass through the biological membranes and it also protects the active herbal components from degradation. Hence, this phospholipid-complex-technique can enable researchers to deliver the phytoconstituents into systemic circulation by using certain conventional dosage forms like tablets and capsules. This review highlights the unique property of phospholipids in drug delivery, their role as adjuvant in health benefits, and their application in the herbal medicine systems to improve the bioavailability of active herbal components. Also we summarize the prerequisites for phytosomes preparation like the selection of type of phytoconstituents, solvents used, various methods employed in phytosomal preparation and its characterization. Further we discuss the key findings of recent research work conducted on phospholipid-based delivery systems which can enable new directions and advancements to the development of herbal dosage forms.

## Introduction

Although, phytoconstituents have been utilized as medicines since ages, the drug delivery system used for administering these herbal medicines to the patients remained antiquated leading to sub therapeutic efficacy in the treatment of a disorder or ailment.^1^ Under such circumstances novel drug delivery system (NDDS) can be of utmost beneficial in improving the efficacy of the herbal compounds or extracts with concomitant reduction in side effects of active compounds.^2^ Usually, the bioavailability of orally administered drugs is governed by several factors like solubility across gastrointestinal transit, release from dosage form, gut permeability, metabolism and drug liability to efflux.^3^ A majority of the plant constituents, specifically phenolic compounds, are hydrophilic and possesses major hurdles like poor lipid solubility, large molecular size, and degradation in the gut owing to their liable nature to acidic and enzymatic environment which limits their application in the therapeutic usage.^4^ To improve the bioavailability of these water soluble molecules in the body, phospholipids based drug delivery system has been found to be promising.^5^ Owing to their better biocompatibility and biodegradability, natural materials like polysaccharides, proteins and phospholipids have gained much attention.^6,7^

Indeed, in 1989, Indena an Italian pharmaceutical and neutraceutical company have developed phospholipid complexation technique by chemically reacting polyphenolic plant actives with phospholipids containing phosphatidylcholine (PC) and later patented the technology with the name PHYTOSOME®.^8^

Phospholipid complex-technique, can serve as a potent drug delivery system for increasing therapeutic index which encapsulates, plant actives. In fact, these complexed actives are safer than its original form^1^ and can even serve as a better targeting agents to deliver these encapsulated agents at specific sites there by proving promising candidates in various medical fields for improving health aspects. This technique can be applied for both herbal and conventional dosage forms and are often known as phytosome and pharmacosome respectively.^9^ The major difference between phytosome and pharmacosomes are shown in Table 1. As the name “phytosome” suggests that it is mainly utilized for plant based molecules which have poor solubility in biological system. Apart from these, it has wider benefits like minimizing toxicity, diminution in dose, and increase in retention time, which makes them potent vehicles for the drug delivery of various drug molecules.^10^

**Table 1:** Major difference between Pharmacosomes and Phytosomes

**Particulars**	**Phytosome**	**Pharmacosomes**
Bond	Weak bond; Hydrogen bond	Strong bond; Covalent bond
Time	More tedious and time consuming	Less tedious
Nature	Amphiphilic	Amphiphilic
Drug leakage	Less	No
Entrapment efficacy	Low	High
Membrane fluidity	Occurs and Controls rate of release	Doesn’t occur and doesn’t control rate of release
Lipid drug interaction	Yes	Yes
Drug release	By Bilayer diffusion, surface desorption, or degradation	By hydrolysis
Stability	Less Stable; less shelf-life	Highly stable; greater shelf-life
Mode of administration	Topical and oral	Topical oral, intravascular

## Prerequisite for phyto phospholipid complex formation

1. Standardized extract or an active phytoconstituent
2. Carrier Phospholipid
3. Solvent

## Standardized extract or an active phytoconstituent selection

1. Basically, either active constituents or standardized extract were selected for phospholipid complex formulation. However, natural products after isolation and purification may lead to a limited or total loss of specific biological activity^11^ so, in such cases whole plant extracts are selected. Usually, phospholipid complex formulations are prepared according to weight basis for standardized extract, whereas molar ratios for active constituent.
2. Selection of plant extract depends on its phytochemical (such as polyphenols, triterpenoids, tannins, alkaloids and saponins) and pharmacokinetic profile. Usually they have multiple ring molecules which are too large to be absorbed by simple diffusion and have low permeability across the cellular lines of the intestine.^12^
3. A drug which contains an active hydrogen atom like –COOH, -OH, -NH_2_, -NH etc., which have the ability to form hydrogen bond between the drug and N-(CH_3_) of PC molecules.^9^
4. Any drugs which possess π electrons can be formulated into different complexes with phospholipid molecules.^13^
5. Both hydrophilic and lipophilic actives can be complexed to improve bioavailability.

## Phospholipids and their importance

In general, fats, phospholipids, and steroids are different types of lipids present in the body and perform various functions. Among them, phospholipids which are major components of cell membranes also serve as a vehicle, thus making the design of drug delivery systems more flexible, and are suitable for the body needs.^14^ Phospholipids are bio friendly and offer various advantages such as formulation flexibility and the choice of different NDDS based on the intended use.^15^ Phospholipids are lipids containing phosphorus, a polar portion and non-polar portion in their structures.^16^

A human biological membrane constitutes different classes of phospholipids, like phosphatidylethanolamine (PE), phosphatidylinositol (PI), phosphatidylcholine (PC), phosphatidic acid (PA), and phosphatidylserine (PS).^17^ PC possess two neutral tail groups and a positive head group which contains an oxygen atom in the phosphate group that has a strong tendency to gain electrons, while nitrogen to lose electrons, a rare molecular characteristic that makes PC miscible in both water and lipid environments.^18^

Earlier “Lecithin” is a word which created perplexity in researchers for identification but later on it was clearly discussed by Wendel.^19^ In commercial perspective, lecithin refers to PC, PE, PS, PI and other phospholipids. But in historical point of view lecithin includes lipids which contains phosphorous obtained from brain and egg. However, scientifically lecithin refers to PC.

## Phospholipid source and its additional benefits as adjuvant

Phospholipids are obtained from both natural and synthetic source. Phospholipids are widely found in plants and animals, and the main sources are vegetable oils soya bean, sunﬂower seed, rapeseed, and cotton and animal tissues include e.g. egg yolk and bovine brain.^18^

Most of the literature suggests the use of soya bean phosphatidylcholine while few others have used egg lecithin, in the preparation of phytophospholipid complex.^20^ In fact, phospholipids are one of the most abundantly present lipid fractions in biological membranes and can form bilayers and act as amphipathic molecules.^21^ After oral administration of phospholipids, they are absorbed to a great extent and reach the peak plasma concentration within 6 hours.^22^ FDA and German Cancer Research Centre, Heidelberg stated that Soy phosphatidylcholine has no carcinogenicity and no risk in formation of tumour.^23,24^ Additionally, PC is said to have varied advanced beneficial properties like hepatoprotective activity,^25^ nutritional supplement to support brain health,^26^ role in membrane fluidity,^27^ shows superior host defences (like enhancing NK cell activity and phagocytosis),^28^ excellent emulsifying activity,^29^ major component of the gastric mucosa lining of the stomach protecting from ulcer,^30^ precursor for acetylcholine,^31^ reducing serum cholesterol,^32^ improving the perception of taste and smell,^33^ recuperate fatigue^34^ and even in nourishing skin.^35^

## Solvents

In phospholipid complexation technique the selection of solvent depends on the solubility of both drug and phospholipids. In fact, literature suggests the use of both aprotic or protic solvents and even many others revealed the use of mixture of solvents for better solubility. Most of the aprotic solvents like diethyl ether, dichloromethane, dioxane, chloroform and n-hexane were recently replaced with ethanol which is safer than the former ones. Table 2 summarizes various solvents used by different researchers.

**Table 2:** Recent works on phytosome, method employed, solvents used and its merit

**Author and year of publication**	**Different phospholipid complex’s**	**Technique employed**	**Types of solvents used**
Junaid K *et al* 2014	Luteolin–phospholipid complex	solvent evaporation Quality by Design employed	ethanol
Shalini S *et al* 2015	Phytosome complex of Methanolic extact of Terminalia Arjuna (TBE)	Salting out	Methylene chloride and methanol ( 6:1) n-hexane
Zahra H *et al* 2015	Rutin-loaded Nanophytosomes	Solvent evaporation method Thin layer hydration method	a mixture of methanol and chloroform(1:4).
Saoji *et al* 2015	Phospholipid-Based Complex of Standardized Centella Extract	salting out	Ethanol, n-hexane
Jun H *et al* 2015	Rosmarinicacid (RA) –phospholipid complex	solvent evaporation	anhydrous ethanol.
Amisha V *et al* 2016	pomegranate extract-phospholipid	Spray drying	equal volumes of dioxane and methanol,
Alisha Pereira *et al* 2015	Phyllanthus emblica extract phospholipid complex	Solvent evaporation technique	dichloromethane or methanol as solvent
Fei L *et al* 2015	Echinacoside phospholipid complex	solvent evaporation method 1:3 molar ratio	tetrahydrofuran
Tianhong Z *et al* 2015	oleanolic acid-phospholipid complex	solvent evaporation method 1:1 molar ratio	anhydrous ethanol
Jin C *et al* 2015	Epigallocatechin Gallate-phospholipid Complex	solvent evaporation method	ethanol.
Maryana *et al* 2015	silymarin–phospholipid complexes	solvent evaporation method 1:5	ethanol

## Methods of phospholipid complex preparation

The following methods were employed in the phospholipid complex preparation like, solvent evaporation, salting out- anti solvent precipitation, mechanical dispersion methods.

## Solvent evaporation method

This technique, involves addition of both the phytoconstituents and PC in a flask containing organic solvent. This reaction mixture is kept at an optimum temperature usually 40 ^o^C for specific time interval of 1 hr to attain maximum drug entrapment in the phytosomes formed. The organic solvent is then removed using rotary evaporator. Thin film phytosomes are sieved by using 100 mesh sieves, and stored in desiccators for overnight.^36,37^ The resultant phytosomes are stored in a light resistant amber colored glass bottle, ﬂushed with nitrogen at room temperature to attain stability.^38^

## Salting out anti solvent precipitation method

In anti solvent precipitation method both the selected phytoconstituents and PC are taken in flask containing a common organic solvent and the mixture is refluxed at desired temperature for specific period on a magnetic stirrer. The solution is later concentrated and anti-solvent like n-hexane is added.^39^ Phospholipid complex will form as a precipitate which is further filtered under vacuum and stored in an air tight amber colored glass container.

## Mechanical dispersion method

In this method, the lipids dissolved in organic solvent are brought in contact with aqueous phase containing the drug.^40^ Initially, pc is dissolved in diethyl ether which is later slowly injected to an aqueous solution of the phytoconstituents to be encapsulated. The subsequent removal of the organic solvent under reduced pressure leads to the formation of phyto-phospholipid complex.

Novel methods for the phospholipid complex preparation includes super critical fluids (SCF), which include gas anti-solvent technique (GAS) compressed anti solvent process (PCA), supercritical anti solvent method (SAS).^41,42^

## Optimization and Characterization techniques

Consistency in Phospholipids complex depend on various factors like drug to phospholipid ratio, experimental duration of time**,** temperature, rotation per minute RPM (in solvent evaporation method), type of drying method employed. All these parameters are optimized statistically through quality by design (QbD).^43-46^

Usually, the characterization of phospholipid complex requires multiple techniques to authenticate and validate its size, shape and morphology.

### Visualization

Phospholipid complexes are visualized either by SEM or TEM. Both these techniques employ electrons as source to produce high resolution images. In SEM, when a sample is bombarded with a beam of electrons, it emits three kinds of electrons, primary backscattered electrons, secondary electrons, and auger electrons and X-rays. In SEM, Secondary electrons provide the surface topography, backscattered electrons give information about the atomic number and X-rays furnish information about the elemental composition of the sample.^47^ Besides, TEM employs transmitted electrons and reveal surface topography with clear detailed internal structure and crystallographic information of the sample.^48^

### Entrapment efficiency

The drug entrapment efficiency is calculated by performing ultra centrifugation technique where certain amount of phyto-phospholipid complex is weighed equivalent to the quantity of herbal drug that is encapsulated and added to phosphate buffer (pH 6.8) later the contents were stirred on a magnetic stirrer for specific period of time and allowed to stand for one hour. Later on, the clear liquid is decanted and centrifuged at 5000 rpm for 15 minutes; supernatant is filtered through 0.45µ Whatman filter paper and finally, absorbance is measured by using UV or HPLC.

The drug entrapment percentage (%) is calculated by using the following formula: Drug entrapment (%) = Actual amount determined/Theoretical amount present.^49^

### Crystallinity and Polymorphism

Differential scanning calorimetry (DSC) and X-ray diffraction (XRD) are mostly adopted techniques for characterization of crystallinity and polymorphism. In phospholipid complex, DSC interactions are typically observed as the elimination of endothermic peaks, appearance of new peaks, changes in peak shape and its onset, peak temperature/melting points and relative peak area, or enthalpy. On the other hand, in XRD phospholipid complex is characterized either by complete absence or disappearance or reduction in the intensity of large diffraction peaks corresponding to its crystalline drug.^50^

***Vesicle stability:*** It mainly depends on particle size, poly disparity index (PDI) and zeta potential commonly measured by single instrument Malvern Zeta Sizer, Malvern Instruments, Malvern, UK.^51^ PDI refers to width of a particle size distribution while zeta potential is a measure of its surface potential. Generally, in phospholipid complex, size may varies from 50 nm to a few hundred µm but the PDI value > 0.5 are unstable and indicating that the sample has a very broad size^52^ distribution and contain large particles or aggregates that may slowly sediment and whereas samples with zeta potential value > ± 30 mV are considered to be stabile.^52^

### Spectroscopic conformation

Phyto-phospholipid complexation and molecular interactions in solution are studied by employing different spectroscopic techniques like ^1^H-NMR, ^13^C-NMR, ^31^P-NMR, and IR spectroscopy. Usually, complex formation and interactions is associated with some characteristic signals like changes in chemical shift and line broadening in NMR spectra’s and with appearance of new bands in IR spectra.^53^

## Phospholipid complex and their absorption

Phospholipid complexes may be absorbed from the GIT through enterocyte based transport, and drug transport to the systemic circulation via intestinal lymphatic system which has widespread network throughout the body. The major advantage of lymphatic transport is to bypass the first-pass metabolism and applicable for targeted drug delivery.^54,55^

**Figure 1:**
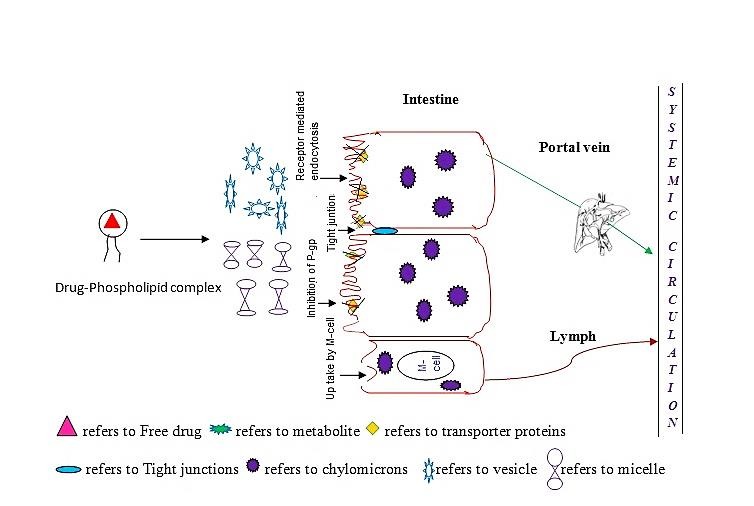
Schematic diagram representing the possible mechanism of phyto-phospholipid complex absorption

After oral administration, the possible mechanism by which lipids affect drug bioavailibity is shown in Figure 1. Schematic diagram representing the possible mechanism by which pyto-phospholipid complex entry into the intestine from unstirred water layer is both by direct solubilization through enterocytes or by endocytosis, paracellular transport in lateral tight junctions, inhibiting drug efflux by blocking transporter proteins, formation of chylomicron production and entering lymphatic port.

The possible mechanisms suggested by various researchers include Yeap *et al* suggested that lipids are absorbed through enterocytes.^55^Stremmel *et al* revealed paracellular transport of phosphotidylcholine through lateral tight junctions.^56^ Holm *et al* claimed that lipid emulsifier such as bile salts and excipients can Inhibit drug efflux by blocking transporter proteins like P-gp and/or CYP 450.^57^ Jain *et al* as stated that drug absorption through endocytosis.^58^ Peng et al stated that possibility of lipoprotein / chylomicron production and entering through lymphatic port.^59^

## Recent advancements

Phospholipid complex technology is adapted for both active and passive targeting in cancer therapy. Lie *et al* formulated a surface functionalised phytosomes of mitomycin C with folate-PEG in targeting HeLA cell and exhibited superior efficacy under both *in-vitro* as well as *in-vivo* conditions.^60^ Whereas sabzichi *et al* demonstrated luteolin phytosomes which sensitized MDA-MB breast cancer cell to doxorubicin and thus assisted in passive targeting of drugs towards these cells.^61^

Xia *et al* formulated a novel drug–phospholipid complex enriched with micelles for 20(S)-protopanaxadiol (PPD) by using a solvent-evaporation method, employing phospholipid and labrasol. The results revealed 64 times better water solubility of 20(S)-protopanaxadiol.^62^

Ochi *et al* has co-delivered novel pegylated nano-liposomal herbal drugs of silibinin and glycyrrhizic acid (nano-phytosome) to target hepatocellular carcinoma (HCC) cell line (HepG2). *In-vitro* study reveals that nano-liposome encapsulation of silibinin with glycyrrhizic acid enhanced the biological activity and stability of silibinin, and synergized the therapeutic effect of silibinin with glycyrrhizic acid.^63^

Abdelkader *et al* formulated a novel phytosomal technology for ocular delivery of L-carnosine by combining hyaluronic acid (HA) hydrogel and phospholipid by using solvent evaporation preparation method consequently, showed enhancement in rheological characteristics, spreading ability, sustained drug permeation, and tolerability characteristics for potential ocular delivery of L-carnosin.^64^

Mazumder *et al* developed *in -vitro* skin permeation of sinigrin from its phytosome complex. The *in vitro* study revealed controlled and sustained release of sinigrin from the phytosome complex. Results suggested that there is possibility of utilizing this sinigrin-phytosome complex for optimal deliver of sinigrin to the skin.^65^

Angelico *et al* encapsulated silybin-phospholipid complex into a liposome forming a supramolecular aggregate and named it as Phyto-Liposomes by employing reverse-phase evaporation method and demonstrated its ability to internalize in human hepatoma Huh7.5 cells and exhibited three hundred folds more potent pharmacological activity.^66^

Amelia *et al* developed a self-nano emulsifying drug delivery system (SNEDDS), based on the phospholipid -complex technique. Initially, Ellagic acid EA phospholipid complex (EAPL) was prepared by anti solvent method, later on SNEDDS were prepared by determining its solubility in different oils, surfactants and co-surfactants and revealed potent *in-vitro* drug release and *ex-vivo* permeation and served as a promising approach for the formulation development of other drugs or phytoconstituents which have limited bioavailability.^67^

## Conclusion

The phyto-phospholipid complexation technique has emerged as an imperative tool in improving bioavailability of herbal drugs. This technique has effectively solved the issue and has offered a preparation of herbal drugs with sufficient lipid penetrability at higher concentration and sustained therapeutic levels in plasma with a slower rate of elimination. A more quantity of active drug has been made available at the site of action. However, it has few limitations which includes a lack of mechanistic connection, quantitative guidance regarding when the lipid-based systems will enhance bioavailability and how to formulate drugs to achieve the desired impact. If we can address the above said limitations, these formulations can serve as a promising candidates for enhancing health regimen of an individual.

## Acknowledgments

Authors acknowledge the grant provided by the Department of Science and Technology, Govt. of India for conducting the research work.

## Ethical Issues

Not applicable.

## Conflict of Interest

The authors declare no conflict of interests.
